# The impact of a social norms approach on reducing levels of misperceptions around smokefree hospital entrances amongst patients, staff, and visitors of a NHS hospital: a repeated cross-sectional survey study

**DOI:** 10.1186/s12889-018-6231-x

**Published:** 2018-12-11

**Authors:** Scott Crosby, Diane Bell, Gerard Savva, Becky Edlin, Bridgette M. Bewick

**Affiliations:** 1 Wakefield Council, Wakefield, WF1 2EB UK; 2 Public Health England, Leeds, LS1, 4PL UK; 3 Magpie Creative Communications Ltd, Leeds, LS2 9NG UK; 4 Leeds Institute of Health Sciences, School of Medicine, University of Leeds, Leeds, UK

**Keywords:** Public health, Tobacco control, Smokefree, Social norms

## Abstract

**Background:**

Smoking is a cause of avoidable morbidity and mortality. In the United Kingdom (UK) the national smoking ban inside hospital buildings is widely adhered to. There is a perception it has led to smokers congregating around hospital entrances (Selbie D. 2016, It’s time for a truly smokefree NHS. Public Health Matters Blog. Public Health England). Efforts to shift social norms and create positive smokefree environments might be strengthened by delivering social norms messages. This study explored the impact of a social norms approach campaign to reduce levels of misperceptions surrounding support for smokefree hospital entrances.

**Method:**

Repeated cross sectional study design. Staff, patients, and hospital visitors at Pinderfields National Health Service (NHS) Hospital (Wakefield, United Kingdom (UK)) completed a survey before and after implementation of a public health social norms campaign (*n* = 481 surveyed before; *n* = 459 surveyed after). The main outcome measure was difference between perceived and reported levels of support for smokefree hospital entrances.

**Results:**

There were high levels of support for smokefree hospital entrances. The majority of participants agreed that patients (*n* = 849, 90% agreed), staff (*n* = 863, 92% agreed), and visitors (*n* = 850, 90% agreed) should not smoke in the hospital entrance.

Participants underestimated the proportion of others who self-reported keeping the entrance smokefree. Over 90% of respondents reported not smoking in the hospital entrance, but the perception was that between 50 to 75% of hospital staff, patients, and visitors did not smoke in the hospital entrance.

The mean percentage of hospital staff, patients, and visitors who respondents thought did not smoke in entrances was higher for respondents responding after, compared to those responding before, the campaign. There was an overall significant effect of time on attitudes towards smoking in the entrances; in all instances the mean percentage of hospital staff, patients, and visitors the participants believed agreed that hospital entrances should be smokefree was higher for those responding after, compared with before, the campaign.

**Conclusions:**

People hold misperceptions of the proportion of people who choose to smoke in the hospital entrance. The social norms approach campaign was associated with a strengthening of positive social norms. Such campaigns should be considered by Trusts as one evidence-based based tactic to denormalise smoking, increase support for smokefree policies.

**Electronic supplementary material:**

The online version of this article (10.1186/s12889-018-6231-x) contains supplementary material, which is available to authorized users.

## Background

Smoking is a major cause of inequalities in health [1, 2] and an avoidable cause of morbidity and mortality in the United Kingdom [3, 4]. The prevalence rate of smoking in England is 15.5% in the adult population [5]. Nationally the cost of smoking to the NHS has been calculated to be between £2.5 billion [6, 7] and £5.2 billion [8].

Evidence suggests that smoke-free legislation has been effective in protecting people from the harmful effects of second-hand smoke exposure while in public places and buildings [9–11]. There is however evidence for mixed support for smoke-free policies in some NHS settings (e.g. psychiatric services) [12]. Internationally, countries report mixed results after implementing smokefree policies within hospital settings. For example, Greece struggled to implement their 2002 ban of smoking in enclosed places [13].

The introduction and implementation of the smokefree legislation in England in 2007 has had public health benefits by helping smokers to quit, reducing cigarette consumption and smoking prevalence [6]. While the national smoking ban inside buildings is understood and adhered to, there is a perception this has led to staff, patients and visitors smoking at hospital entrances and within hospital grounds [14, 15]. Unintended consequences of smoking bans that inadvertently see a concentration of smokers at entrances or perimeters remain a public health concern [16, 17].

National Institute for Health and Care Excellence (NICE) guidance PH48 on smoking and acute, maternity and mental health services includes recommendations on developing, implementing and communicating, smokefree policies; this includes smokefree grounds [18]. However, only one in sixteen hospitals have completely enforced the ban on smoking on hospital premises with calls for trust boards to be held to account to enforce smoke free hospital policies and support quit attempts [14, 19].

In March 2017 the national planning document ‘Next steps on the NHS Five-Year Forward View’ set a deadline for all of the NHS estate to be smokefree by 2019/20 [20]. In July 2017 the Department of Health published ‘Towards a Smokefree Generation – a Tobacco Control Plan for England’ [6] which included a commitment to make all mental health inpatient service sites smokefree by 2018. It recommended that Public Health England and NHS England work together to implement NICE guidance on helping pregnant smokers to quit; to provide access to training for all health professionals on how to help patients to quit; and that NHS Trusts encourage smokers using, visiting and working in the NHS to quit, with the goal of creating a smokefree NHS by 2020.

Widening tobacco control policies could help shift social norms about the acceptability of exposing others to second-hand smoke and changing cultural attitudes and norms to smoking.

Reducing smoking requires action at an individual and environmental level. Intervention is most effective when it is part of a portfolio of work aimed at denormalisation of smoking (e.g. [21]). For the NHS, this portfolio includes formal policies on smoking (e.g. smokefree hospital grounds) and awareness raising campaigns designed to change the social acceptability of smoking within a NHS premises. Research into normative misperceptions has given rise to a new form of intervention, known as the social norms approach [22, 23]. The premise of this approach is simple; if individuals’ misperceptions of behaviours and/or attitudes can be corrected then the perceived social support for them to engage in positive health behaviours (e.g. keeping hospital entrances smokefree) will be increased and their own engagement in the positive behaviour will increase.

The social norms approach has been used to increase health behaviours in schools, colleges, and universities but there remains a paucity of research investigating the feasibility and effectiveness of implementing social norms approach campaigns in healthcare settings [23]. The current study investigated the outcomes associated with a social norms approach campaign designed to reduce misperceptions of the number of people who smoke in the hospital entrance and who support smoking in the hospital entrance.

The current study aimed to investigate differences in responses between hospital staff, patients, and visitors who completed a survey before a social norms campaign with those who completed the same survey after the implementation of the social norms campaign. More specifically, the current study aimed to investigate:

- Differences observed before and after the campaign in perceptions of the proportion of hospital staff, patients, and visitors who do not smoke in the hospital entrance.
- Differences observed before and after the campaign in perceptions of the proportion of hospital staff, patients, and visitors who agree that the hospital entrance should be smokefree.

## Method

### Design

The changes in perceptions of support for smokefree hospital entrances study has a repeat cross sectional design. Survey questions were asked before and after the implementation of a public health social norms campaign (see supplementary file for survey questions). The campaign was designed to reduce levels of misperceptions surrounding support for smokefree hospital entrances amongst hospital patients, visitors, and staff. The survey investigated self-reported actual and perceived social norms associated with smoking in hospital entrances and on hospital grounds. Serafin et al. reported the results of the secondary analysis of the free text responses provided by participants [15].

### Setting

Pinderfields Hospital is a NHS hospital based in Wakefield (United Kingdom). It is part of The Mid Yorkshire Hospitals NHS Trust that provides community, acute (hospital-based treatment) and specialist health services to around half a million people living in the Wakefield and North Kirklees areas. The hospital is approximately one mile from Wakefield City Centre. The hospital has one main entrance used by staff, patients, and visitors. The double-door entrance is step-free with a canopy that offers some protection from the weather. In 2006 Pinderfields Hospital implemented a smokefree hospital grounds policy that meant smoking was no longer permitted anywhere on the ground. This included at the entrance, in car parks, on paths or and on roads. The sign at the road entrance to the hospital included the words ‘Welcome to a smoke-free hospital’ and the glass front near the entrance to the hospital building displayed a no-smoking icon. In 2012 a minority of staff, patients, and visitors continued to smoke on hospital grounds. Staff and patients complained of smoke entering through windows opened above the entrance. The Mid-Yorkshire Hospitals NHS Trust launched the social media campaign in an effort to make the hospital grounds completely smokefree. The timing of the activity was as follows: before campaign survey 10th–18th September 2012, implementation of campaign 26th November-17th December 2012, after campaign survey 17th – 21st December 2012.

### Participants

All hospital patients, visitors, and staff on the premises of Pinderfields hospital during data collection periods were eligible to participate. The sample was one of convenience. One thousand individuals were approached. In total *n* = 963 questionnaires were returned (96% return rate). Of these 23 were excluded (*n* = 20 age < 18 years, *n* = 3 < 50% completion). Therefore *n* = 940 completed questionnaires were eligible for inclusion in the analysis (i.e. 94% response rate). The current analysis incudes data collected from *n* = 481 participants before implementing the campaign (*n* = 164 patients, *n* = 143 hospital visitors, *n* = 163 hospital staff, unknown/other *n* = 11), and data collected from *n* = 459 participants after the campaign (*n* = 157 patients, n = 143 hospital visitors, *n* = 156 hospital staff, other n = 3).

### Ethics

This study received approval from Research and Development (R&D) (Mid Yorkshire NHS Trust). The Trust approved the study as an audit and evaluation of smoking behaviour on Pinderfields hospital grounds. Permissions were granted via NHS Wakefield.

All participants were provided with information to enable informed consent. Completion and return of the pen and paper questionnaire indicated consent. Individuals had the right to refuse to participate. All questionnaires were completed anonymously and once surveys were returned it was not possible for data to be withdrawn.

### Procedure

Convenience sampling was undertaken. Data collection was organised to ensure that all accessible areas of the hospital building and grounds were covered. Paper surveys were distributed throughout the hospital and grounds (e.g. reception areas, hospital wards, outpatient service waiting areas, administrative staff areas, staff coffee rooms, canteen areas, hospital shuttle bus queue). Questionnaires were distributed face-to-face by a researcher and self-completed by the participant. Where possible questionnaires were returned to/collected by the researcher. Where this was not possible participants were asked to return completed questionnaires to reception. In total 1000 surveys were distributed and 940 (94%) were completed, returned, and included in the current analysis. The incentive for the completion and return of each survey was a donation of £1 to a local charity paid on their behalf. Three choices of charity were given: The Mid Yorkshire Hospitals NHS Trust Charitable Fund, Wakefield Hospice and Macmillan Nurses.

### Main outcomes measures

The main outcome measure was the difference between perceived and reported levels of support for smokefree hospital entrances. Perceived and reported levels of support were measured using questions adapted from existing social norms approach surveys. Participants were asked to indicate their level of agreement (5 point Likert scale, strongly agree to strongly disagree) with the following statements: ‘Hospital patients should not smoke in hospital building entrances’; ‘Hospital staff should not smoke in hospital building entrances’; ‘Visitors should not smoke in hospital building entrances’. Participants were then asked to estimate how many people (hospital patients, hospital staff, and hospital visitors) agree that ‘Pinderfields Hospital entrances should be a place where people don’t smoke’; responses were recorded using a visual analogue scale (no patients thru to all patients). Secondary outcome measures included perceived and reported behaviour (i.e. smoking in hospital entrances and/or grounds). The measure of self-reported behaviour asked participants to indicate the statement that best described them. Their choice of statements was: I am a non-smoker; I am a smoker but do not smoke anywhere on hospital grounds; I do not smoke in the entrances to the hospital building, but I do/would smoke elsewhere on the grounds; I only smoke in the entrances to the hospital building, but would not smoke elsewhere on the grounds; I smoke on hospital grounds, this includes in entrances to the hospital building (see Additional file 1 for a copy of the questionnaire).

### Intervention

**Fig. 1:**
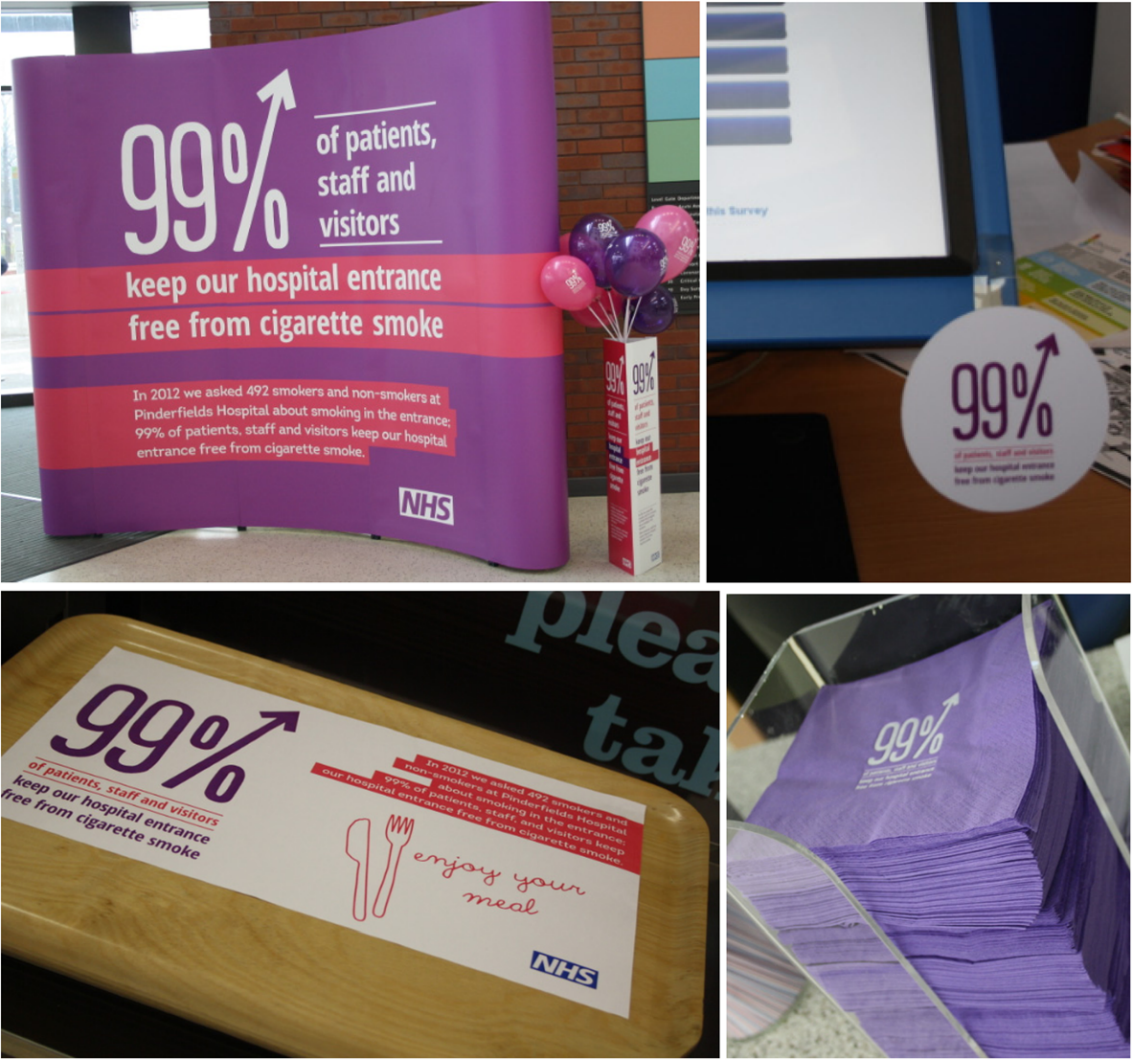
Examples of intervention material displayed on and around Pinderfields hospital

Returned pre-campaign questionnaires were used to derive the marketing message. In total 485 questionnaires provided self-reported data on whether or not participants smoked in the hospital entrance. Of these 478 (i.e. 98.6%) reported not smoking in hospital entrances. The intervention message was created by a marketing agency. For the purposes of the campaign a decision was made to keep the language as simple as possible and to avoid academic jargon. The campaign message did not therefore make a distinction between self-reported and actual behaviour. Social norms marketing strategies were used to disseminate the message that ‘99% of patients, staff, and visitors keep our hospital entrance free from cigarette smoke’. The message was displayed and promoted using a variety of print media displayed throughout the hospital. The print-media and hospital locations were chosen to be highly visible to the target audience of staff, visitors, and patients in areas of high footfall and around the hospital. Print media included: expo and pull up banners, posters, wobblers, stickers, pins, canteen napkins and tray lining, café barriers, wraps on bollards outside the hospital entrance, peelable window stickers and leaflets on staff payslips (see Fig. 1 for examples).

### Analysis

Analysis of Variance (ANOVA) was used to examine the effects of time (before vs. after campaign) on perceptions of smokefree entrance behaviour and perceptions of attitudes towards smokefree entrances of staff, patients, and visitors. ANOVA was deemed appropriate for this ordinal data as studies have shown that Visual Analogue Scales have interval and ratio properties and so can be treated as numerical [24]. ANOVA was also used to examine the effects of time (before vs. after campaign) on self-reported smoking behaviour in the entrances by staff, patients, and visitors.

## Results

Mean age of participants 49.12 years (SD = 16.07). The majority of participants were white/white British (*n* = 846, 90%), with 6% Asian/Asian British (*n* = 52) and < 1% Black/African/Caribbean/Black British (*n* = 7). In total 68% (*n* = 636) were female. The majority of participants reported being non-smokers (*n* = 794, 85%).The overwhelming majority of participants reported not smoking in hospital entrances (*n* = 916, 97%). Not smoking on hospital grounds was also reported as a majority behaviour (*n* = 867, 92%).

ANOVA revealed an overall significant effect of time across the three variables investigating perceptions of smoking in entrances (F(3, 910) = 8.44, *p* < 0.001). With significant differences between the two data collection points (before and after) in the perceived percentage of patients (F (1, 912) = 22.21, p = < 0.001), staff (F(1,912) = 11.78, *p* = 0.001), and visitors (F(1, 912) = 12.50, *p* < 0.001) respondents perceived did not smoke in the hospital entrances. In all instances the mean of the percentage of respondents thought did not smoke in entrances was higher for respondents responding after, compared to those responding before, campaign implementation (see Table 1).

**Table 1:** Perceived attitudes and behaviour of patients, staff, and visitors towards smokefree hospital entrances reported before and after the social norms marketing campaign

		Before (*n* = 469)	After (*n* = 465)	Overall (*n* = 934)	
Percentage of [A/B/C] I think do not smoke in entrances					
[A] Patients	Mean (SD)	46 (24)	54 (28)	50 (26)	*p* < 0.001
	Median (IQR)	50 (27, 64)	50 (31, 80)	50 (29, 72)	
[B] Staff	Mean (SD)	68 (26)	74 (26)	71 (27)	*p* = 0.001
	Median (IQR)	75 (50, 92)	83 (54, 98)	78 (50, 96)	
[C] Visitors	Mean (SD)	48 (25)	54 (28)	51 (27)	*p* < 0.001
	Median (IQR)	50 (27, 67)	50 (31, 81)	50 (29, 75)	
		Before (*n* = 471)	After (*n* = 467)	Overall (*n* = 938)	
Percentage of [A/B/C] I think agree hospital entrances should be smokefree					
[A] Patients	Mean (SD)	61 (21)	69 (24)	65 (23)	*p* < 0.001
	Median (IQR)	61 (50, 77)	73 (50, 90)	68 (50, 83)	
[B] Staff	Mean (SD)	69 (23)	75 (23)	72 (23)	*p* < 0.001
	Median (IQR)	74 (50, 89)	81 (53, 95)	77 (50, 93)	
[C]Visitors	Mean (SD)	62 (22)	68 (24)	65 (23)	*p* < 0.001
	Median (IQR)	64 (50, 79)	73 (50, 89)	69 (50, 84)	

ANOVA revealed an overall significant effect of time across the three attitudes towards smoking in the entrance variables (F(3, 9.14) = 10.72, *p* < 0.001). With significant differences between the two data collection points (before and after) in the perceived percentage of patients (F (1, 916) = 29.14, p = < 0.001), staff (F(1,916) = 15.10, p < 0.001), and visitors (F(1,916) = 12.25, *p* < 0.001) respondents perceived agreed that hospital entrances should be smokefree. In all instances the mean of the percentage respondents thought agreed that hospital entrances should be smokefree was higher for those responding after, compared with before, campaign implementation (see Table 1).

ANOVA revealed no overall effect of time on self-reported smoking in entrances (F(2,912) = 0.60, *p* = 0.55). The proportion of staff, patients, and visitors self-reporting smoking in entrances before and after the campaign was 98% (*n* = 457) and 98% (*n* = 445) respectively. There was a significant effect of category of participant (i.e. staff, patient, or visitor) (F(2,913) = 17.55, *p* < 0.001). Post-hoc pair-wise comparisons revealed staff reported significantly lower levels of smoking in entrances (< 1%) compared to visitors (3%) or patients (2%) (see Table 2). No significant interaction between time and category of participant was observed (F(2,913) = 0.75. *p* = 0.47).

**Table 2:** Self-reported behaviour of patients, staff, and visitors before and after the social norms marketing campaign

		Before (*n* = 464)	After (*n* = 455)	Overall (*n* = 919)
Percentage of [A/B/C] who report not smoking in the hospital entrance ^a^				
[A] Patients	n (%)	158 (99%)	153 (98%)	311 (98%)
[B] Staff	n (%)	162 (99%)	155 (100%)	317 (100%)
[C] Visitors	n (%)	137 (97%)	137 (96%)	274 (97%)
Overall [A,B, & C]	n (%)	457 (98%)	445 (98%)	902 (98%)

^a^excludes *n* 14 participants whose participant category was other/unknown

Results illustrate high levels of support for smokefree hospital entrances. The majority of participants agreed that patients (before *n* = 435, 91% agreed; after *n* = 414, 90% agreed), staff (before *n* = 437, 92% agreed; after *n* = 426, 93% agreed) and visitors (before *n* = 431, 92% agreed; after *n* = 419, 92% agreed) should not smoke in the hospital entrance. Although the majority supported smokefree hospital grounds, support for not smoking on hospital grounds was lower. More specifically, between 60 and 72% agreed that patients (before *n* = 288, 60% agree; after *n* = 315, 69%), staff (before *n* = 300, 64% agree; after *n* = 330, 72% agree) and visitors (before *n* = 298, 63%; after *n* = 329, 72%) should not smoke on hospital grounds.

## Discussion

The majority (98%) of patients, visitors, and staff reported not smoking in the hospital entrance. The perception amongst respondents completing the survey before the campaign was that 46, 68, and 48% of patients, staff and visitors respectively did not smoke in the hospital entrance. This was despite the fact that the majority of respondents reported not smoking and believed that the entrance to the hospital should be smokefree.

The social norms marketing campaign was designed to promote the message that the majority of people reported keeping the entrance smokefree. This campaign, was associated with a reduction in the percentage of people participants’ believed smoked at the hospital entrances. Specifically, we observed a small yet significant increase of people believing that patients, staff and visitors kept the entrances smokefree. The social norms theory predicts that highlighting that the majority of patients, staff, and visitors report not smoking in the hospital entrance would encourage those that do smoke in the entrance to conform to the ‘norm’ and make the hospital entrance smokefree.

Like many NHS hospitals in England, Pinderfields Hospital has a no smoking policy. The current study highlighted misperceptions of the social norm as one of the challenges faced when implementing and enforcing no smoking polices. Specifically, the perception that the majority of people will smoke in entrances being at odds with the majority of people reporting supporting smokefree hospitals and reporting that they themselves keep the entrance smokefree. The results from this study provided staff at the hospital with evidence that the majority of people report not smoking in the hospital entrance and showed support for keeping the entrance smokefree. This may encourage other people to conform to the ‘norm’ and either not smoke or smoke elsewhere and give staff the confidence to challenge smokers at the entrance knowing that the majority of people agree with keeping the entrance smokefree.

The success of the implementation of the intervention was aided by the team’s ability to build relationships with hospital management and staff. These relationships also enabled researchers to gain access throughout the hospital, this included access to inpatients on the ward. Given access to a wide variety of areas in the hospital building helped to ensure there was equal representation from the three target groups of staff, visitors, and patients.

Due to restrictions on respondents being given a financial incentive to complete the survey a charity donation of £1 was offered for each survey completed. Respondents picked from a choice of three charities, this proved a good recruitment incentive and encouraged engagement as evidenced by the relatively high response rate.

Limitations of the project include the sample being one of convenience rather than randomly selected. While researchers collected data from a variety of locations around the hospital there were areas of restricted access which researchers could not enter and the timing of data collection from each location was one of convenience. It is therefore likely that the sample is not representative of all those present on the hospital grounds during the data collection period. The sample included more women than men (68% vs 32%), and a self-reported smoking prevalence of 15%. In 2012 25% of adults 16 years or over in the Wakefield area reported smoking [25]. It is not possible to ascertain if the sample was representative of those present on the hospital grounds during the data collection period or if the demographic profile is a result of sampling biases introduced as a result of the decision to use a convenience sample. In addition, the current project used data from two cross-sectional data collection points. This before-and –after design means changes across time cannot be attributed with confidence to the campaign. Data was collected using cross sectional surveys at two timepoints, the design would be strengthened had we been able to follow participants across time. Respondents smoking status was self-reported and we were unable to verify smoking status or smoking behaviour using objective measures. Despite responses being anonymous it is possible that social desirability bias influenced participant’s willingness to identify as someone who smoked in hospital entrances and on hospital grounds. Social desirability is likely to have been felt most acutely by hospital staff. The design would have been strengthened from objective observations of smoking behaviour at and around the hospital entrance. The campaign message ‘99% of patients, staff, and visitors keep our hospital entrance free from cigarette smoke’ may have led to some participants believing that the data had been collected using objective measures of smoking at the hospital entrance. A more accurate campaign message may have been ‘99% of patients, staff, and visitors report keeping our hospital entrance free from cigarette smoke’. The current data can say nothing about how the wording was received nor of the extent to which the distinction between ‘self-report’ and ‘actual’ behaviour is of importance to the intended audience.

Hospitals have a duty to protect the health and wellbeing of staff, visitors, and patients. Public Health England chief executive Duncan Selbie has called on hospital managers for greater leadership on tobacco control [14, 26]. Smoking kills 79,000 people each year in the UK. It was responsible for 474,000 hospital admissions in 2015/2016. In spite of smokefree legislation, over 30% of men and 26% of women still report some exposure to second-hand smoke [27]. People smoking at the entrance to NHS trusts gives a poor impression, leads to increased litter of cigarettes butts and also means that those entering and leaving buildings have to pass through tobacco smoke. For people using secondary care services, smoking is associated with increased risk of complications and delays to recovery [18]. Having a smokefree policy in a NHS hospital which is not adhered to or enforced undermines public health messages around the dangers of smoking.

The social norms intervention at Pinderfields Hospital was valuable in raising the discussion and awareness of keeping the hospital entrance smokefree amongst staff, patients, and visitors. To capitalise on the interest and exposure this campaign has generated it is advisable that other measures are now rolled out to achieve a smokefree hospital included improved signage, staff training, policy revision, enforcement, and a revised communication strategy to ensure the smokefree message is disseminated through all external and internal communication.

## Conclusion

This study showed high levels of support amongst the target audience for keeping hospital entrances smokefree. It also highlighted misperceptions that participants had in relation to other people smoking in the hospital entrance. Despite only 1% of people smoking in the hospital entrance participants believed this figure to be much higher. This apparent misperception could be due to the high visibility of smokers and the lingering tobacco smoke at the hospital entrance. This study should encourage the hospital to maintain and enforce the smokefree policy on the hospital site and encourage the development of other areas to ensure the hospital does become completely smokefree. Social norms messages alone are unlikely to change all smokers’ behaviour. The approach should be combined with smokefree policies, training, and enforcement to help encourage smokers to conform to the behaviour of the majority, which in this case is not smoking in the hospital entrance. This study offers a platform for further work to reinforce the smokefree policy throughout the hospital with all audiences, building on the awareness raised by the social norms campaign.

## Additional file

Additional file 1: Pinderfields hospital survey. (PDF 96 kb)

## Abbreviations

ANOVA: Analysis of variance
NHS: National health service
NICE: National Institute for health and Clinical Excellence
R&D: Research and development
SD: Standard deviation
UK: United Kingdom
