# Supplementary material for: The impact of a social norms approach on reducing levels of misperceptions around smokefree hospital entrances amongst patients, staff, and visitors of a NHS hospital: a repeated cross-sectional survey study
Source: BMC Public Health. 2018 Dec 11;18:1365. doi: 10.1186/s12889-018-6231-x (PMC6288883; doi:10.1186/s12889-018-6231-x)
Supplement: Supplementary file 1 — Pinderfields hospital survey. (PDF 96 kb) [file 12889_2018_6231_MOESM1_ESM.pdf]

Date:

Time:

NHS
Wakefield District

just your best guess?

Pinderfields Hospital Survey 2012

We ask you to find 5-10 minutes to fill in this survey.
Your answers will make a difference to Pinderfields hospital.
By completing it you will ensure your views are listened to.

DONATION TO CHARITY
For every survey completed and returned a donation of £1 will be made to a local charity . Please indicate which charity you would like us to donate to on your behalf:

- ☐ The Mid Yorkshire Hospitals NHS Trust Charitable Fund
- ☐ Wakefield Hospice
- ☐ Macmillan Nurses

Thank You

just your best guess?

Pinderfields Hospital Survey 2012

We are collecting this information so we can understand what people think about smoking around the grounds of Pinderfields Hospital.

IT SHOULD TAKE 5-10 MINUTES TO ANSWER THE QUESTIONS.

- Everything you tell us will be treated in confidence
- Please answer each question honestly
- There are no right or wrong answers
- Please do not ask anybody else when you answer the questions. We are interested in your opinion.
- Follow the instructions for each question carefully.
- When you have completed the survey please give it back to the person you gave it to you or put in it inside the envelope provided and hand in to:

Pinderfields General Office
(open Monday to Friday 9am - 5pm).
Outside these times please hand into a member of staff at the reception desk.

Thank you for agreeing to take part in this survey.

In this questionnaire we will ask about smoking in hospital entrances and hospital grounds.

By this we mean:

Hospital entrances are any entrances that lead into the hospital building.

Hospital grounds are any area within the boundry of the hospital site, including car parks.

SECTION 1: About you

1. Are you male or female? *Please tick one box* ☐ Male ☐ Female

2. How old are you? *Please write your age in the box* ☐ Years

3. Which of the following best describes the reason for your being at Pinderfields Hospital today?
Please tick ☑ *a suitable box.*

☐ I am a hospital in-patient
☐ I am a hospital out-patient
☐ I am visiting (or here with) a patient
☐ I am Pinderfields Hospital staff, *please specify below*
  ☐ Clinical
  ☐ Domestic
  ☐ Technical
  ☐ Administration/Management
  ☐ Other (please specify) ....
☐ I am visiting (or here with) Pinderfields Hospital staff
☐ Other (please specify) ....

4. What is your ethnic group?

We are asking this question so we can understand more about what people from different backgrounds think. *Please tick* ☑ *one box.*

☐ I'd prefer not to say

White
☐ English / Welsh / Scottish / Northern Irish
☐ Irish
☐ Gypsy or Irish Traveller
☐ Any other White background (please specify)
....

Mixed/multiple ethnic groups
☐ White and Black Caribbean
☐ White and Black African
☐ White and Asian
☐ Any other Mixed / multiple ethnic background (please specify)
....

Asian / Asian British
☐ Indian
☐ Pakistani
☐ Bangladeshi
☐ Any other Asian background (please specify)
....

Black / African / Caribbean / Black British
☐ Caribbean
☐ African
☐ Any other Black / African / Caribbean background (please specify)
....

Other ethnic group
☐ Any other ethnic group (please specify)
....

SECTION 2: Your own opinions and behaviour

The following questions ask about what you think other people at Pinderfields Hospital do in relation to smoking habits in and around the hospital grounds.

Some questions ask about staff, some ask about patients, and some ask about visitors.

If you are unsure what we mean by hospital entrances or grounds please refer to page 2.

5. Do you agree or disagree with these statements about smoking in the hospital entrances?
Please tick ☑ one box on each line of this question

	Strongly Agree	Agree	Neither Agree or Disagree	Disagree	Strongly Disagree
HOSPITAL PATIENTS **should not smoke** in hospital building entrances	☐	☐	☐	☐	☐
HOSPITAL STAFF **should not smoke** in hospital building entrances	☐	☐	☐	☐	☐
VISITORS **should not smoke** in hospital building entrances	☐	☐	☐	☐	☐

6. Do you agree or disagree that people should not smoke on hospital grounds?
Please tick ☑ one box on each line of this question

	Strongly Agree	Agree	Neither Agree or Disagree	Disagree	Strongly Disagree
HOSPITAL PATIENTS **should not smoke** on hospital grounds	☐	☐	☐	☐	☐
HOSPITAL STAFF **should not smoke** on hospital grounds	☐	☐	☐	☐	☐
VISITORS **should not smoke** on hospital grounds	☐	☐	☐	☐	☐

The following question asks about you smoking tobacco (including cigarettes and roll-ups) on Pinderfields Hospital grounds.

7. Which of the following BEST applies to you? *Please tick ☑ one box only.*

☐ I am a non-smoker.

☐ I am a smoker but do not smoke anywhere on hospital grounds.

☐ I do not smoke in the entrances to the hospital building, but I do/would smoke elsewhere on the grounds.

☐ I only smoke in the entrances to the hospital building, but would not smoke elsewhere on the grounds.

☐ I smoke on hospital grounds, this includes in entrances to the hospital building.

SECTION 3: The opinions of others in relation to smoking

These questions ask about **what you believe other people** who come to Pinderfields Hospital **think** in relation to smoking in and around the hospital grounds.

It doesn't matter if you really don't know the answer – it's just your best guess.

STATEMENT 1 **"Pinderfields Hospital entrances should be a place where people don't smoke."**

8. a) How many **HOSPITAL PATIENTS** do **you think would agree** with this statement?

Mark your 'X' at any point on the line.

0% ——————————————————— *100%*

no patients | about half of patients | all patients

b) How many **HOSPITAL STAFF** do **you think would agree** with this statement?

Mark your 'X' at any point on the line.

0% ——————————————————— *100%*

no staff | about half of staff | all staff

c) How many **HOSPITAL VISITORS** do **you think would agree** with this statement?

Mark your 'X' at any point on the line.

0% ——————————————————— *100%*

no visitors | about half of visitors | all visitors

STATEMENT 2 **"Pinderfields Hospital grounds should be a place where people don't smoke."**

9 a) How many **HOSPITAL PATIENTS** do **you think would agree** with this statement?

Mark your 'X' at any point on the line.

0% ——————————————————— *100%*

no patients | about half of patients | all patients

b) How many **HOSPITAL STAFF** do **you think would agree** with this statement?

Mark your 'X' at any point on the line.

0% ——————————————————— *100%*

no staff | about half of staff | all staff

c) How many **HOSPITAL VISITORS** do **you think would agree** with this statement?

Mark your 'X' at any point on the line.

0% ——————————————————— *100%*

no visitors | about half of visitors | all visitors

SECTION 4: The behaviour of others in relation to smoking

These questions ask about **what you think other people** who come to Pinderfields Hospital **do** in relation to smoking and around the hospital grounds.

It doesn't matter if you really don't know the answer – it's just your best guess.

STATEMENT 3 **How many of these people do you think smoke in the entrances to Pinderfields Hospital?**

10 a) **HOSPITAL PATIENTS**

Mark your 'X' at any point on the line.

0% ... *100%*

no patients | about half of patients | all patients

b) **HOSPITAL STAFF**

Mark your 'X' at any point on the line.

0% ... *100%*

no staff | about half of staff | all staff

c) **HOSPITAL VISITORS**

Mark your 'X' at any point on the line.

0% ... *100%*

no visitors | about half of visitors | all visitors

STATEMENT 4 **How many of these people do you think smoke on the grounds of Pinderfields Hospital?**

11 a) **HOSPITAL PATIENTS**

Mark your 'X' at any point on the line.

0% ... *100%*

no patients | about half of patients | all patients

b) **HOSPITAL STAFF**

Mark your 'X' at any point on the line.

0% ... *100%*

no staff | about half of staff | all staff

c) **HOSPITAL VISITORS**

Mark your 'X' at any point on the line.

0% ... *100%*

no visitors | about half of visitors | all visitors

If you have any other comments you would like to add, please put them here.

THANK YOU FOR COMPLETING THIS SURVEY

ALL THE INFORMATION WILL BE KEPT CONFIDENTIAL

PLEASE GIVE IT BACK TO THE PERSON WHO GAVE IT TO YOU
or put it in the envelope provided and return it to:

Pinderfields General Office
(open Monday to Friday 9am - 5pm).

Or hand into a member of staff at
the reception desk.
